# Mismatch repair deficiency reshapes glioblastoma through immune suppression rather than hypermutation

**DOI:** 10.1172/JCI203730

**Published:** 2026-03-16

**Authors:** Andrew Li, Thomas K. Sears, Craig M. Horbinski

**Affiliations:** Department of Laboratory Medicine and Pathology, Mayo Clinic, Jacksonville, Florida, USA.

## Abstract

Mismatch repair (MMR) deficiency is classically associated with microsatellite instability, a high tumor mutational burden (TMB), and sensitivity to immune checkpoint blockade in cancer. In this issue of the *JCI*, Puigdelloses Vallcorba et al. reported that this paradigm does not hold true in glioblastoma (GBM). Using genetically engineered mouse models, the authors demonstrated that loss of core MMR genes was insufficient to induce hypermutation or improve survival rates with PD-1 blockade. Instead, mouse models of germline MMR deficiency accelerated malignant progression by promoting the immune milieu toward a myeloid cell-dominant and T cell–suppressed tumor microenvironment. Importantly, the imidazotetrazine agent *N^3^*-(2-fluoroethyl) imidazotetrazine (KL-50) bypassed MMR dependence and overcame temozolomide resistance. These findings suggest MMR deficiency in GBM as a driver of immune suppression rather than tumor immunogenicity and carry important implications for therapy selection.

## Connecting MMR loss, hypermutation, and PD-1 sensitivity

Mismatch repair (MMR) proteins such as MSH2, MSH6, MLH1, and PMS2 correct base mismatches and insertion-deletion loops generated during DNA replication. In many cancers, loss of MMR activity leads to microsatellite instability (MSI), accumulation of mutations, and, in certain contexts, the emergence of highly immunogenic hypermutant tumors with robust responses to programmed cell death 1 (PD-1) blockade. This relationship is sufficiently consistent that MMR deficiency has become a tissue-agnostic biomarker for predicting the response to immune checkpoint inhibition (1–3).

Glioblastoma (GBM), however, has challenged this conventional wisdom. Although recurrent GBMs frequently acquire MMR gene alterations after exposure to temozolomide (TMZ), these tumors rarely demonstrate durable benefits from immunotherapy (4). Whether this reflects incomplete MMR loss, insufficient neoantigen formation, or an intrinsically suppressive brain tumor microenvironment was addressed in the current study (5).

## MMR-deficient GBM lacks hypermutation and PD-1–responsive characteristics

Puigdelloses Vallcorba et al. (5) directly tested whether MMR loss itself is sufficient to generate a hypermutant and immunotherapy-responsive GBM. Using replication-competent avian-like sarcoma virus/tumor virus receptor-A–driven (RCAS/Tv-a–driven) mouse glioma models with either germline or somatic deletion of the MMR genes *Msh2* or *Msh6*, the authors found that MMR deficiency did not reliably increase the tumor mutational burden (TMB) or induce MSI on its own. Whole-exome sequencing revealed mutational burdens comparable to those of MMR-proficient tumors, and anti–PD-1 therapy failed to improve survival of MMR-deficient mice.

These findings align with human GBM datasets, in which most tumors harboring single MMR gene mutations remain microsatellite stable and do not exhibit the ultrahypermutant phenotype unless additional defects, such as polymerase mutations (i.e., POLE/POLD1), are present. Thus, in the RCAS/Tv-a–driven GBM model, MMR loss alone was insufficient to generate the immunogenic features seen in other malignancies (4, 6).

## Immune suppression drives deleterious outcomes in MMR-deficient GBM

This study’s main, unexpected insight emerged when the authors distinguished germline from tumor-restricted (somatic) MMR loss in mouse models. Germline deletion of *Msh2* markedly accelerated progression from low-grade glioma to GBM, shortening survival, whereas somatic MMR loss confined to tumor cells did not.

Importantly, this survival disadvantage was not explained by increased tumor cell proliferation or altered differentiation states. Instead, it reflected a profound change in the immune milieu of the tumor microenvironment. Single-cell RNA-seq and spectral flow cytometry revealed expansion of disease-associated myeloid cell populations, including monocyte-derived macrophages and activated microglia, coupled with reduced lymphoid infiltration. CD8^+^ T cells exhibited transcriptional and phenotypic features of exhaustion, including elevated expression of PD-1 and other immune checkpoint molecules.

These data indicate that MMR deficiency exerts its deleterious effects primarily through immune regulation rather than tumor cell–intrinsic mutagenesis. Notably, the aggressive phenotype required MMR loss in nontumor compartments, implicating host immune cells such as microglia and infiltrating macrophages as key mediators specifically in the context of germline MMR deficiency.

## Germline versus somatic MMR loss

The study’s distinction between germline and somatic MMR deficiency provides a unifying explanation for several interesting observations. Germline loss affected not only tumor cells but also microglia, infiltrating macrophages, and lymphocytes, thereby predisposing the tumor ecosystem to immune suppression. In contrast, somatic MMR loss limited to tumor cells preserved an MMR-proficient immune compartment and did not replicate the same adverse outcomes (5).

This distinction has important implications for interpreting MMR alterations in patients. In adult GBM, most MMR mutations arise somatically after TMZ exposure and may not reflect the biology of inherited MMR deficiency. Conversely, in pediatric constitutional MMR deficiency syndromes, gliomas often display extreme hypermutation and responsiveness to immune checkpoint blockade (7). Differences in developmental timing, cell of origin, and cooperating mutations likely determine whether MMR loss manifests primarily as immunogenicity or as immune suppression.

## TMZ resistance reflects loss of MMR-dependent cytotoxicity

Consistent with prior work, both MMR-reduced and MMR-deficient tumors were resistant to TMZ (5). This resistance arose not from immune evasion but from disruption of the MMR-dependent “futile repair” pathway required for TMZ-induced cytotoxicity (4). TMZ treatment of MMR-proficient tumors reduced intratumoral myeloid cell populations but also induced systemic lymphopenia, underscoring its broad effects on dividing cells (5).

In MMR-deficient settings, TMZ failed to exert either a tumoricidal or immune-modulating benefit (5). These findings suggest that the clinical failure of TMZ in MMR-altered GBM reflects a loss of DNA damage recognition rather than an absence of immune engagement.

## KL-50 bypasses MMR dependence and suppresses resistant GBMs

To overcome this limitation, Puigdelloses Vallcorba et al. evaluated *N^3^*-(2-fluoroethyl) imidazotetrazine (KL-50), an imidazotetrazine designed to induce DNA interstrand crosslinks independently of the MMR pathway. KL-50 effectively suppressed tumor growth and extended survival in MMR-proficient, MMR-reduced, and fully MMR-deficient models (8, 9). In fact, our group’s previously published work suggests that MMR-deficient GBM cells are even more sensitive to KL-50 than might be expected, based solely on MMR mechanisms (9). This may be due to the fact that MMR enzymes like MSH2 and MSH6 are also involved in the repair of interstrand crosslinks (10).

Beyond direct cytotoxicity, KL-50 alters the tumor immune landscape, reducing immunosuppressive myeloid cell subsets while increasing CD8^+^ T and NK cell infiltration. These combined effects position KL-50 as a rational therapeutic strategy for TMZ-resistant GBM and highlight the value of targeting DNA repair vulnerabilities without relying on MMR function.

## Implications and future directions

The study by Puigdelloses Vallcorba et al. changes how MMR deficiency should be interpreted in GBM. Detection of an MMR gene aberration alone, in the context of an untreated GBM, should not be interpreted as indicative of hypermutation or immunotherapy responsiveness. Whether MMR loss leads to immune activation or immune suppression depends on which cells are affected and when the loss occurs.

Several questions remain to be investigated. Is this atypical consequence of MMR deficiency specific to GBM, or is it applicable to other tumors? What players in the signaling pathways guide MMR-deficient immune cells toward myeloid skewing and T cell exhaustion? Can these suppressive circuits be reversed? How should patients with MMR-altered GBM be stratified for alkylating agents, DNA crosslinkers, or immunotherapy? How do other therapies perform in the MMR-deficient GBM context? In addition, mouse lifespan and tumor growth characteristics of the model may limit the accumulation of ultrahypermutation characteristics such as those observed in gliomas associated with constitutional MMR deficiency (CMMRD), an aggressive cancer predisposition syndrome characterized by biallelic loss-of-function mutations in MMR genes.

Clinically, these findings argue for caution in applying tissue-agnostic immunotherapy approaches to GBM and instead highlight the potential of therapies that bypass defective DNA repair pathways. KL-50 and related agents represent a promising step in this direction.

Ultimately, Puigdelloses Vallcorba et al. (5) have demonstrated that in GBM, MMR deficiency does not automatically make tumors more immunogenic but instead drives immune suppression. Their work highlights the importance of viewing glioma as a system-level disease shaped by interactions between tumor genetics and host immunity.

## Version 1 03/16/2026

Electronic publication
